# Supplementary figures and images for: Floral Aroma and Pollinator Relationships in Two Sympatric Late-Summer-Flowering Mediterranean Asparagus Species
Source: Plants (Basel). 2023 Sep 10;12(18):3219. doi: 10.3390/plants12183219 (PMC10537274; doi:10.3390/plants12183219)

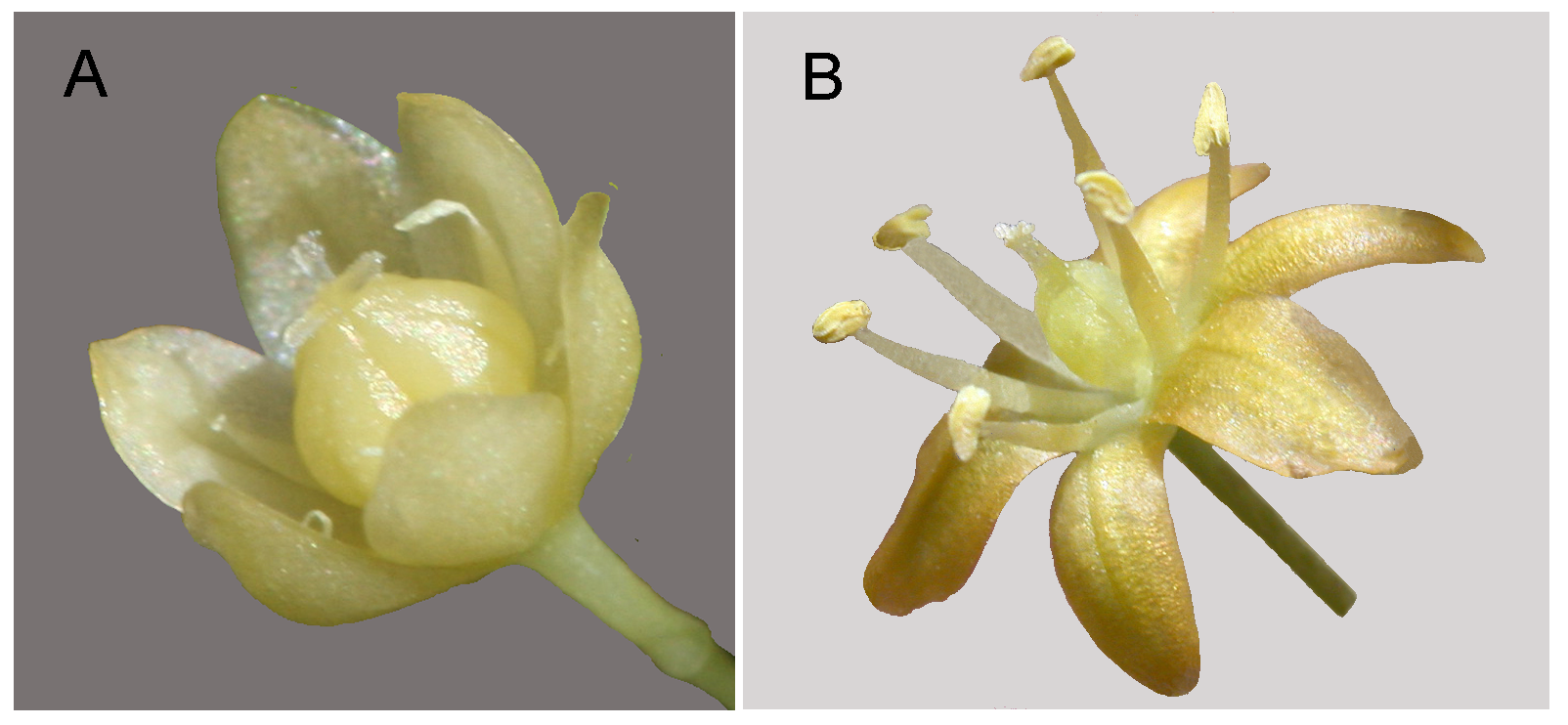

Supplement: Supplementary file 1 [file plants-12-03219-s001.zip › plants-2583263-supplementary/SUPPLEMENTARY/Supplementary S1_Asparagus.tiff]

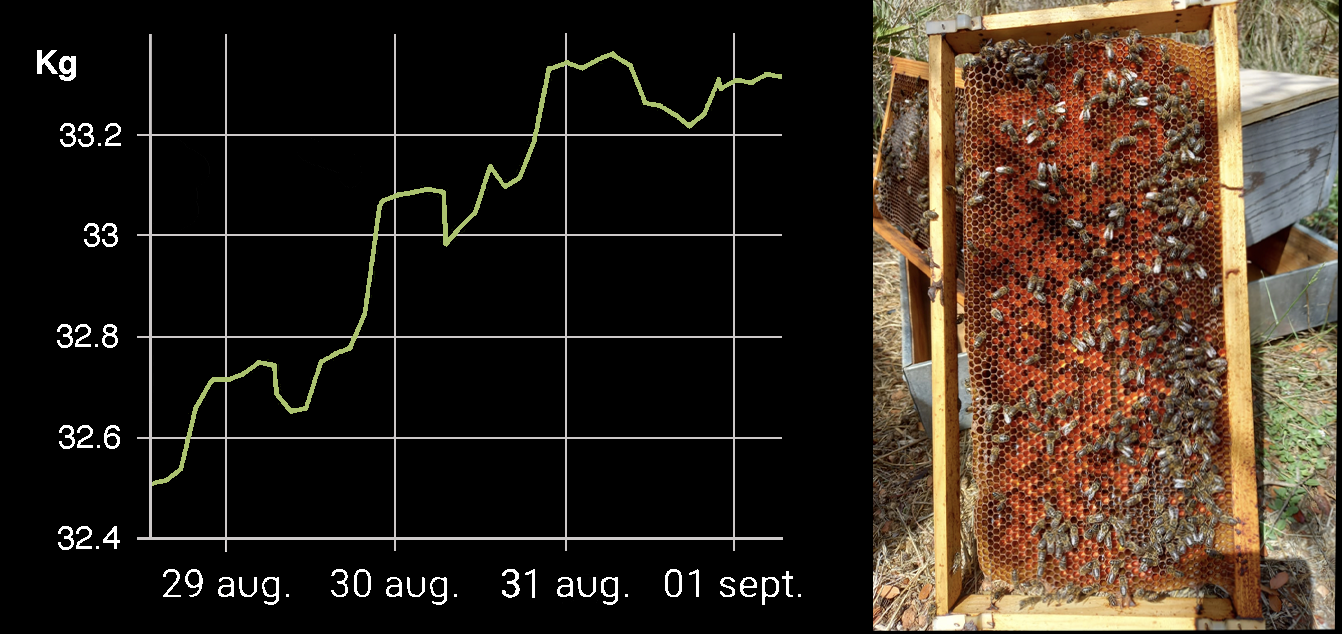

Supplement: Supplementary file 1 [file plants-12-03219-s001.zip › plants-2583263-supplementary/SUPPLEMENTARY/Supplementary S2. A-albus_Pollen hive.tif]

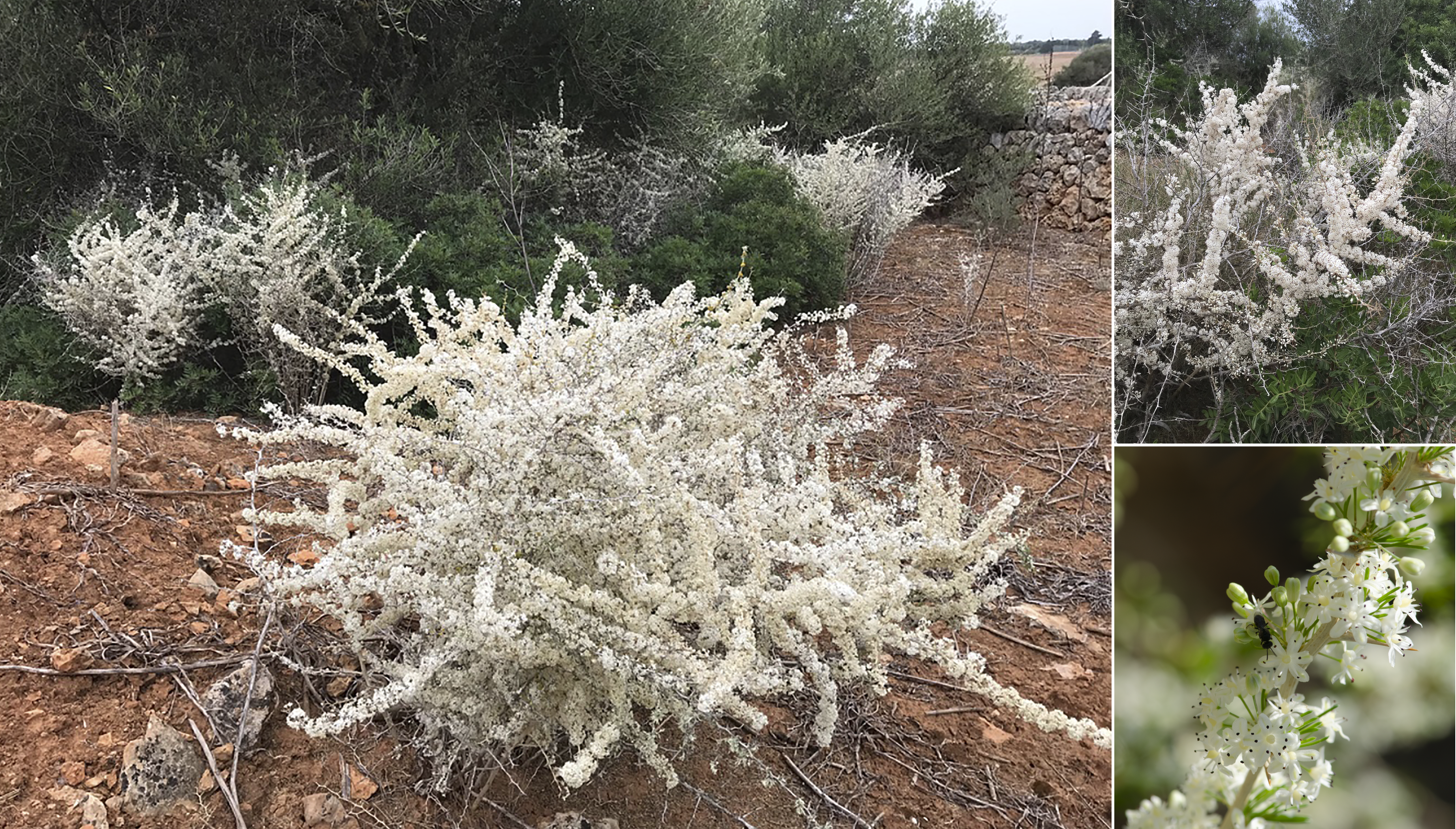

Supplement: Supplementary file 1 [file plants-12-03219-s001.zip › plants-2583263-supplementary/SUPPLEMENTARY/Supplementary S3_Bloom Asparagus albus.tif]
